# Efficacy of Three Different Prophylactic Treatments for Postoperative Nausea and Vomiting after Vitrectomy: A Randomized Clinical Trial

**DOI:** 10.3390/jcm8030391

**Published:** 2019-03-21

**Authors:** Michele Reibaldi, Matteo Fallico, Antonio Longo, Teresio Avitabile, Marinella Astuto, Paolo Murabito, Carmelo Minardi, Vincenza Bonfiglio, Francesco Boscia, Claudio Furino, Robert Rejdak, Katarzyna Nowomiejska, Mario Toro, Gilda Cennamo, Salvatore Cillino, Michele Rinaldi, Tito Fiore, Carlo Cagini, Andrea Russo

**Affiliations:** 1 Department of Ophthalmology, University of Catania, 95123 Catania, Italy; matteofallico@hotmail.com (M.F.); antlongo@unict.it (A.L.); t.avitabile@unict.it (T.A.); enzabonfiglio@gmail.com (V.B.); toro.mario@email.it (M.T.); andrearusso2000@hotmail.com (A.R.); 2 Department of Anaesthesiology, University of Catania, 95123 Catania, Italy; astmar@tiscali.it (M.A.); paolomurabito@tiscali.it (P.M.); minardi.carmelo@virgilio.it (C.M.); 3 Department of Ophthalmology, University of Sassari, 07100 Sassari, Italy; francescoboscia@hotmail.com; 4 Department of Ophthalmology, University of Bari, 70124 Bari, Italy; claudiofurino@gmail.com; 5 Department of General Ophthalmology, Medical University of Lublin, 20079 Lublin, Poland; robertrejdak@yahoo.com (R.R.); katarzyna.nowomiejska@umlub.pl (K.N.); 6 Department of Public Health, University of Naples Federico II, 80131 Naples, Italy; xgilda@hotmail.com; 7 Department of Experimental Biomedicine and Clinical Neuroscience, Ophthalmology Section, University of Palermo, 90127 Palermo, Italy; salvatore.cillino@unipa.it; 8 Department of Ophthalmology, Second University of Naples, 80131 Naples, Italy; michele.rinaldi@unina2.it; 9 Division of Ophthalmology, Department of Surgery and Biomedical Science, University of Perugia, S Maria della Misericordia Hospital, 06129 Perugia, Italy; titofiore@hotmail.com (T.F.); carlo.cagini@unipg.it (C.C.)

**Keywords:** dexamethasone, ondansetron, postoperative nausea and vomiting, vitrectomy

## Abstract

Postoperative nausea and vomiting (PONV) after vitreoretinal surgery may potentially be associated with severe complications, such as suprachoroidal hemorrhage. The purpose of the present multicenter clinical trial (NCT02386059) was to assess the efficacy of three different prophylactic treatments for PONV after vitrectomy under local anesthesia. Patients undergoing primary vitrectomy were randomized to the control arm or to one of the treatment arms (4 mg ondansetron, 4 mg dexamethasone, combination of the two drugs). The primary outcome measure was the proportion of complete response (no nausea, no vomiting, no retching, and no use of antiemetic rescue medication) during 24 h after vitrectomy. Secondary outcomes included the severity standardized score of PONV, postoperative pain standardized score, and rate of ocular and non-ocular adverse events. Baseline demographics of the 1287 patients were comparable between the four arms. The combined therapy group showed a statistically significant lower incidence of PONV compared to the placebo and monotherapy (*p* < 0.001). PONV severity was also reduced in the combination group compared to the others (*p* < 0.001). Postoperative pain scores and adverse events were comparable among the four groups. Combined therapy with dexamethasone and ondansetron was the most effective treatment for reducing the incidence and severity of PONV in patients undergoing vitrectomy under local anesthesia.

## 1. Introduction

Postoperative nausea and vomiting (PONV) after vitreoretinal surgery can be an unpleasant consequence of surgery, and may potentially be associated with severe complications, such as suprachoroidal hemorrhage (SCH) [1]. Suprachoroidal hemorrhage is a very serious condition defined as the presence of blood in the suprachoroidal space, following the rupture of the posterior ciliary arteries or vortex veins [2,3]. It is considered as one of the most devastating complications which may occur in all types of intraocular surgery, including vitreoretinal surgery [2,3,4,5,6,7].

The incidence of SCH after vitrectomy ranges from 0.4% to 1% [6,7]. A recent multicentre study shows that the rate of SCH after vitrectomy was 0.8%, with a permanently impaired visual function in most of the eyes with this complication. The main precipitating factor in these cases was postoperative vomiting [1]. Therefore, the prevention of PONV is of great relevance both for reducing patient discomfort as well as for avoiding serious ocular complications, such as SCH. 

A prospective randomized trial showed that treatment with low-dose droperidol reduced PONV and related complications after vitreoretinal surgery performed under general anesthesia [8]. However, at present, almost all vitreoretinal surgery is performed under local anesthesia, because of quicker patient rehabilitation and the avoidance of possible complications from general anesthesia [9].

To date no study has evaluated the possible effect of therapy in the prevention of PONV after vitrectomy performed under local anesthesia.

Therefore, the aim of this study was to evaluate the efficacy of ondansetron, dexamethasone, and their combination to prevent PONV in patients undergoing vitrectomy under local anesthesia. Postoperative pain and the rate of adverse events were also investigated. 

## 2. Experimental Section

### 2.1. Study Design

This was a prospective, randomized, multicenter, double-blind trial, which was registered at ClinicalTrials.gov (Identifier: NCT02386059) in March 2015.

The study was conducted in compliance with the recommendations described in the Consolidated Standards of Reporting Trials (CONSORT) Statement [10], with the tenets of the Declaration of Helsinki, and the standards of Good Clinical Practice (Table S1). 

Institutional review board approval was obtained at each study site before the enrollment of patients. The study objective, methods, and period, and the expected adverse events were explained to the patients before obtaining their written and informed consent for participation. 

### 2.2. Participants

All consecutive patients that underwent primary vitrectomy under local anesthesia between May 2015 and January 2017 at the Department of Ophthalmology of the University of Catania (Italy), Department of Ophthalmology of the University of Sassari (Italy), and Department of Ophthalmology of the University of Bari (Italy) were assessed for eligibility. The following clinical conditions were considered as inclusion criteria: (1) Retinal detachment; (2) vitreous hemorrhage; (3) dropped lens/IOL; and (4) macular disease, such as macular pucker and macular hole [11]. If a patient had vitrectomy in both eyes, the patient was included in the study for the first vitrectomy.

Exclusion criteria were:

- Patients younger than 18 years;
- an American Society of Anesthesiologists (ASA) physical status > III;
- diabetes mellitus;
- hypersensitivity to study drugs or rescue medication;
- patients suffering from acute or chronic nausea, motion sickness, and/or vomiting;
- a severe hepatic insufficiency (Child-Pugh score > 9);
- a clinically significant or unstable cardiac, respiratory, hepatic, renal, or other major organ system disease or a psychotic illness or depression;
- addiction to illicit substances or alcohol;
- patients who had taken antiemetic within 12 hours prior to surgery;
- chronic corticosteroid treatment;
- pregnancy; and
- subjects who, in the opinion of the investigator, would experience an unacceptable risk from the administration of the study drugs.

### 2.3. Randomization and Treatment

After inclusion in the study, the enrolled patients were randomly assigned, in a 1:1:1:1 ratio, to one of four groups related to prophylactic antiemetic treatment, in a double-blind manner using computer-generated codes placed in sequentially numbered opaque envelopes:

- Group A = placebo, received intravenously (IV) two syringes containing 10 mL of saline, one at the start of surgery and one 15 min before the end of surgery.
- Group B = one syringe of ondansetron, 4 mg diluted to 10 mL IV 15 min before the end of surgery, plus one syringe containing 10 mL of saline at the start of the surgery.
- Group C = one syringe of dexamethasone, 4 mg diluted to 10 mL IV, at the start of surgery plus one syringe containing 10 mL of saline 15 min before the end of surgery.
- Group D = one syringe of 4 mg dexamethasone diluted to 10 mL IV at the start of surgery and one syringe of 4 mg ondansetron diluted to 10 mL IV 15 min before the end of surgery.

Two sets of sealed envelopes were used for both males and females to ensure a uniform gender distribution between the groups. An anaesthetist who was not involved in the treatment opened the envelopes and prepared the prophylactic antiemetic treatment in identical syringes immediately before the start of anesthesia. The patients and staff who made the postoperative evaluations were blinded to the group assignments.

Peribulbar local anesthesia consisted of a mixture of 5 mL lignocaine 2%, 5 mL bupivacaine 0.75%, and 1500 U of hyalase. No additional analgesic or sedative measures were administered during surgery. 

After recruitment and allocation to the prophylactic treatment, all patients who shifted to general anesthesia, or who received any drug which could have an effect on PONV were excluded from the study.

In the recovery room and at 2, 8, and 24 h postoperatively, the patients were checked for the manifestation of nausea and emetic episodes (defined as dry retching or vomiting) and were also asked for nausea and pain assessment by a blinded observer. Additionally, the medical records were inspected. Patients rated nausea using a 4-point scale (none, mild, moderate, or severe) [8]. Following institutional guidelines, 10 mg of intramuscular metoclopramide was administered when the patient experienced nausea or had 2 emetic episodes within 2 hours. This was followed by 4 mg ondansetron IV when metoclopramide alone was ineffective.

Similarly, patients rated pain using a 4-point verbal rating scale (no pain, mild, moderate, or severe) [12]. When a patient complained of moderate to severe pain, 1 g paracetamol IV was administered. This was followed by 30 mg ketorolac IV (a maximum of 120 mg/day), when paracetamol alone proved ineffective. 

To classify the severity of PONV during the 24-h observation period in 4 degrees, we used a standardized scoring algorithm that has been used and described in similar trials. Briefly, no PONV defined the absence of any emetic symptoms and nausea during the entire study period. Mild PONV described the occurrence of mild nausea or one episode of vomiting if caused by an exogenous stimulus (e.g., drinking or movement). Moderate PONV was designated when the patient vomited up to 2 times or experienced nausea that required a rescue antiemetic therapy only once. Patients were classified as having severe PONV if they suffered more than two emetic episodes or necessitating more than one dose of a rescue antiemetic [8,13].

Likewise, postoperative pain severity during the 24-h observation period was also categorized in 4 degrees. Briefly, no pain described the absence of any painful symptom during the 24-h study period. Mild pain was defined as the occurrence of mild pain at least once during the study period. Moderate pain was defined as the occurrence of moderate pain at least once during the study period with or without administering only paracetamol rescue therapy. Severe pain was defined as the occurrence of severe pain at least once during the study period that required administration of analgesics or the occurrence of moderate pain that did not resolve after paracetamol rescue therapy and required a further drug.

Eye examination, including slit lamp and fundus examination and Goldmann tonometry, was performed on the first day after surgery and, also, when PONV or pain occurred. 

Additionally, patient demographic, systemic, preoperative, and intraoperative data were recorded and analyzed, along with possible side effects of the administration of the study drugs (e.g., akathisia/restlessness, drowsiness, dizziness, headache). 

### 2.4. Study Objectives

The primary outcome of the study was to evaluate the proportion of patients with complete response (absence of PONV: No nausea, no vomiting, no retching, and no use of antiemetic rescue medication) during the first 24 h after vitrectomy.

Secondary outcome measures were a standardized score of nausea and/or vomiting severity if PONV occurred within 24 h and a standardized pain score during the same post-operative period.

Non-ocular and ocular adverse events occurring during the study period were considered as secondary outcomes and were recorded. High IOP was defined as an IOP value ≥ 30 mmHg, whereas hypotony was an IOP value ≤ 6 mmHg. Treatment of IOP rise included oral acetazolamide 250 mg at IOP ≥ 30 mmHg, additional topical combination of timolol 0.5% and dorzolamide 2% at IOP ≥ 35 mmHg, as well as additional topical brimonidine 0.2% at IOP ≥ 40 mmHg.

### 2.5. Statistical Analysis

The sample size (at least 300 eyes for each group) was determined from the results of our preliminary data (30% in untreated patients, and 8% in patients treated with dexamethasone and ondansetron) to detect, with an alpha of 0.05 and 90% power (one-tailed), a 20% difference in the rate of complete response (absence of PONV: No nausea, no vomiting, no retching, and no use of rescue medication). To allow for a percentage of unsuccessful treatment, at least 330 eyes per group were randomized. The rate of patients with complete response (absence of PONV) after vitrectomy in the four groups was compared by the KRUSKAL-WALLIS test; if significant, multiple comparisons between groups were performed by Dunn-Bonferroni pairwise comparison test.

Chi square test was used to compare the rate of high IOP in PONV cases and non PONV cases.

*p* values < 0.05 were considered as statistically significant. Statistical analysis was conducted using the SPSS 11.5.1 soft package for Windows (SPSS, Inc., Chicago, IL, USA).

## 3. Results

### 3.1. Patients’ Disposition and Baseline Characteristics

**Figure 1:**
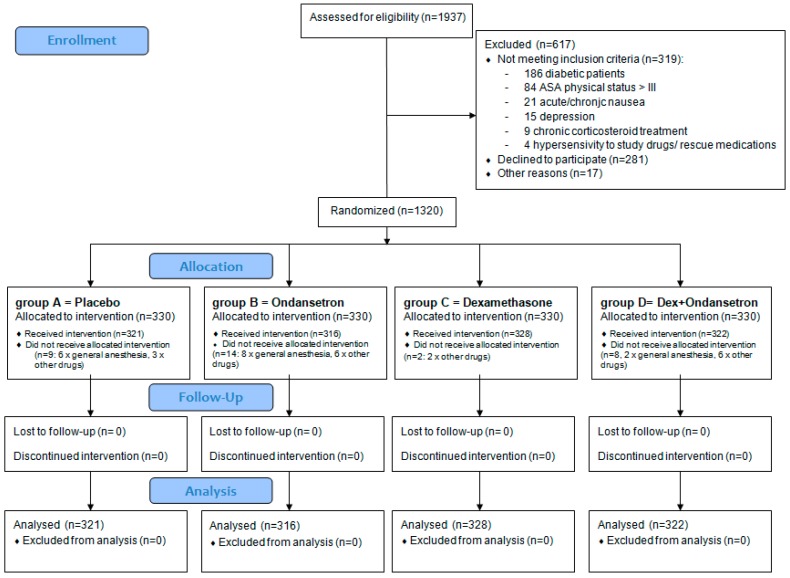
Recruitment flowchart.

The recruitment flowchart is displayed in Figure 1. Data from 1287 patients were analyzed.

Baseline demographic and systemic characteristics along with preoperative and intraoperative data are summarized in Table 1. There were no statistically significant differences between the four groups for all the variables analyzed (demographic, systemic, preoperative, and intraoperative data).

### 3.2. Primary Outcome Measures

The primary outcome was the proportion of patients with complete response (absence of PONV: No vomiting, no retching, and no use of antiemetic rescue medication) during the first 24 hours after surgery (Table 2). 

A significant difference was seen among groups (KRUSKAL-WALLIS test *p* < 0.001).

The combination group had the greatest rate of complete response (95.96%, 309 of the 322 patients), which was significantly higher compared to the ondansetron group (80.38%, 254 of 316 patients, *p* < 0.001), to the dexamethasone group (80.79%, 265 of the 328 patients, *p* < 0.001) and to the placebo group (71.96%, 231 of the 321 patients, *p* < 0.001).

The rate of complete response in the ondansetron group and dexamethasone group was higher than in the placebo group (*p* = 0.036 and *p* = 0.023, respectively), with no difference between the two monotherapy groups (*p* = 1).

PONV was more frequent in females in three of the four groups, without significant differences among the groups (Kruskall-Wallis) (Table 3).

PONV was more frequent in patients with arterial hypertension in three of the four groups (Table 4), without significant differences among the groups (Kruskall-Wallis).

### 3.3. Secondary Outcome Measures

Standardized score of nausea and/or vomiting severity if PONV occurs.

The KRUSKAL-WALLIS test shows a statistically significant difference in PONV standardized score among the four groups during the first 24 hours after surgery (*p* < 0.001).

Pairwise comparison showed a higher PONV intensity score (mild = 62, moderate = 23, severe = 5) in the placebo group than in the ondansetron group (mild = 46, moderate = 16; *p* = 0.028), and the dexamethasone group (mild = 45, moderate = 18; *p* = 0.020). There was no significant difference between the two drugs (*p* = 1). The best results were found in the combination group (mild = 11, moderate = 2, *p* < 0.001 vs. placebo group). Combined therapy had better results compared to monotherapy (*p* < 0.001) (Table 5).

### 3.4. Pain Score during the 24 h Post-Operative Period

The KRUSKAL-WALLIS test showed no significant difference in the pain score among the four groups during the first 24 hours after surgery (*p* = 0.080) (Table 5).

### 3.5. Adverse Events

Non-ocular and ocular adverse events are shown in Table 6. No serious non-ocular adverse events were reported during the study period. There was no statistically significant difference in non-ocular adverse event rates among the four groups. (*p* > 0.05). Six serious ocular adverse events were reported throughout the study period: Four SCH cases, of which two were in the placebo group, one in the dexamethasone group and one in the ondansetron group; two cases of retinal detachment, of which one was in the dexamethasone group and one in the placebo group. There was no statistically significant difference in the ocular adverse event rates among the four groups (*p* > 0.05). Additionally, high IOP was also studied considering PONV onset: High IOP was registered in 4.4% of PONV cases and in 2.5% of cases without PONV (*p* = 0.132).

## 4. Discussion

In this study, combined administration of dexamethasone and ondansetron proved to be the most effective prophylactic treatment for the prevention of PONV after vitrectomy performed under local anesthesia.

PONV represents a threatening event during the perioperative period following vitreoretinal surgery, which could irremediably jeopardize visual and anatomical outcomes as it has been associated with the onset of suprachoroidal hemorrhage and intraocular bleeding [1,14].

The incidence of PONV after vitreoretinal surgery has been reported to be roughly 60% when performed under general anesthesia [14,15] and up to 50% under local anesthesia [13].

Thus, the prevention of such a concerning event is necessary to provide patients with the sharpest visual outcome.

A prospective randomized trial studied the prevention of PONV after vitrectomy under general anesthesia, comparing droperidol and dolasetron [8]. The results showed that low-dose droperidol reduced the incidence and the severity of PONV compared to placebo whereas dolasetron did not [8]. However, droperidol has been associated with malignant proarrhythmogenic effects, with a consequent “black box warning” issued by the Food and Drug Administration (FDA) for all doses of droperidol [16]. Furthermore, nowadays, local anesthesia is the mainstream anesthetic practice when it comes to vitreoretinal surgery because of its advantages, such as the avoidance of general anaesthetic risks, good postoperative analgesia, and faster postoperative rehabilitation [9].

General anesthesia is reported to have an 11-fold increased risk for PONV compared to regional anesthesia [17]. In particular, ocular surgery has been associated with an additional risk factor related to the oculoemetic reflex triggered during this type of surgery [8,13].

In the present trial, all patients enrolled underwent vitrectomy under local anesthesia. No other supplementary analgesic or sedative drugs were given during surgery, such as midazolam or opioids, due to their possible influence on the pathophysiological and neurochemical mechanism of PONV [18,19].

The most commonly used antiemetic drugs in the perioperative setting for PONV prevention are Serotonin type 3 (5-hydroxytryptamine type 3 or 5-HT3) receptor antagonists and corticosteroids, which have been given after ocular surgery as well [20]. The former drugs act by antagonizing 5-HT3 receptors both peripherally on vagal afferents and centrally in the area postrema. In particular, ondansetron is the most diffused 5-HT3 receptor antagonist with a typical dosing of 4 mg for the prevention of PONV, and higher doses may be used for the treatment of nausea and vomiting associated with chemotherapy [18].

Combined administration of dexamethasone of 4 mg at the beginning of surgery and ondansetron of 4 mg before the end of surgery is supposed to be the most suitable multimodal approach for preventing postoperative nausea and vomiting [21].

Both ondansetron and dexamethasone present low rates of adverse events. Ondansetron is considered well tolerated, its most common side effect is a headache [22]. In 2012, the FDA recommended not to exceed 16 mg of ondansetron in a single dose due to risk of QT prolongation, and, subsequently, the 32 mg single IV dose has no longer been marketed [23]. However, such a high dose is only administered for chemotherapy-induced nausea and vomiting. This suggests its safety when given in lower doses for postoperative nausea and vomiting [24]. Side effects of dexamethasone are postoperative hyperglycemia and wound infections, which become questionable when a single dose of 4 mg of dexamethasone is administered [18,25].

In this prospective randomized clinical trial, dexamethasone and ondansetron, alone and in combination, were tested for the first time as prophylactic treatment of PONV after vitreoretinal surgery performed under local anesthesia, also evaluating postoperative pain and the rate of adverse events.

Our results demonstrated that both dexamethasone and ondansetron are effective in reducing the incidence and severity of PONV compared to placebo, but the most effective treatment is the combination therapy of these two drugs, which produced a highly significant difference when compared to the placebo group and a significant difference when compared to single drug administration.

Xiang et al. [13] recently conducted a randomized clinical trial to assess the analgesic and sedative efficacy of dezocine and midazolam in vitrectomy under local anesthesia, also analysing their effect on PONV as a secondary outcome. Their results showed that midazolam significantly reduced the PONV rate compared to the placebo group [13]. However, midazolam is a sedative drug, which has been associated with severe hemodynamic and respiratory side effects [26,27].

The incidence of PONV reported by Xiang et al. in the placebo group was 60% [13], much greater than that shown by our results. This discrepancy could to be related to the different methodologies used in the two trials. In particular, the exclusion criteria listed by Xiang et al. [13] did not take into account a condition of chronic or acute nausea, whereas our study relied on strict exclusion criteria to minimize any confounding factors.

Our results also confirm the low rate of adverse events and tolerability of both dexamethasone and ondansetron. The most common non-ocular adverse event recorded was a headache, whose rate was slightly greater in the ondansetron group and the combination group, even if there was no significant difference between groups.

Regarding the possible association between high IOP and PONV onset, our findings showed no difference in high IOP among the four groups and, overall, any association between high IOP and PONV was found.

The present study has some limitations. First, the study was not powered to assess the safety of the study drugs, the aim being to evaluate the effectiveness of the prophylactic treatments. Therefore, we recorded only the rate of adverse events. Second, both postoperative nausea and pain were evaluated by using a 4-point verbal rating scale, which has been considered slightly less sensitive compared to a visual analogue scale [28]. Nonetheless, considering patient discomfort after eye surgery in being analyzed repeatedly through a visual analogue scale, and also taking into account that some authors did use the verbal rating scale for the same purpose [8,13], we decided to use the verbal rating scale.

## 5. Conclusions

In summary, the combination of ondansetron and dexamethasone was seen to be the most effective treatment for the prevention of PONV after vitreoretinal surgery performed under local anesthesia, also presenting a low rate of adverse events. The association of these two drugs may be recommended as the first choice prophylactic treatment for patients scheduled for vitrectomy under local anesthesia.

## Figures and Tables

**Table 1:** Baseline demographic and systemic characteristics, preoperative and intraoperative data.

Variables	Placebo (*n* 321)	Ondansetron (*n* 316)	Dexamethasone (*n* 328)	Ondansetron + Dexamethasone (*n* 322)	*p*
**Demographics**					
Mean ± SD age (years)	66 ± 7	66 ± 10	67 ± 8	66 ± 9	0.344
Male sex, No. (%)	160 (49.8)	157 (49.6)	164 (50.0)	162 (50.3)	0.999
**Systemic**					
Hypertension, No. (%)	140 (43.6)	146 (45.5)	142 (43.2)	139 (43.1)	0.850
Cerebral Stroke, No. (%)	7 (2.1)	4 (1.2)	6 (1.8)	9 (2.7)	0.575
Cardiovascular diseases, No. (%)	31 (9.6)	26 (8.2)	30 (9.1)	23 (7.1)	0.680
Antiplatelet agents/anticoagulants No. (%)	109 (33.9)	99 (31.3)	102 (31.0)	114 (35.4)	0.592
**Ophthalmic**					
Mean ± SD axial length (mm)	24.9 ± 1.6	25.1 ± 1.8	25.2 ± 1.8	25.0 ± 1.7	0.143
Mean ± SD preoperative IOP (mmHg)	14.5 ± 1.9	14.3 ± 1.3	14.6 ± 1.7	14.4 ± 1.5	0.105
Preoperative pseudophakic/aphakic, No. (%)	139 (43.3)	141 (44.6)	129 (39.3)	144 (44.7)	0.468
**Operative**					
Rhegmatogenous retinal detachment, No. (%)	181 (56.3)	183 (57.9)	187 (57.0)	190 (59.0)	0.916
Macular hole, No. (%)	26 (8.0)	25 (7.9)	28 (8.5)	26 (8.0)	0.619
Epiretinal membrane, No. (%)	90 (28.0)	88 (27.8)	90 (27.4)	85 (26.3)	0.967
Dropped lens/IOL, No. (%)	10 (3.1)	8 (2.5)	12 (3.6)	11 (3.4)	0.865
Others, No. (%)	14 (4.3)	12 (3.7)	11 (3.3)	10 (3.1)	0.841
Combined vitrectomy and phaco, No. (%)	118 (36.7)	120 (37.9)	122 (37.1)	114 (35.4)	0.924
23 gauge vitrectomy, No. (%)	247 (76.9)	239 (75.6)	252 (76.8)	254 (78.8)	0.806
25 gauge vitrectomy, No. (%)	69 (21.4)	73 (23.1)	70 (21.3)	64 (19.8)	0.804
27 gauge vitrectomy, No. (%)	5 (1.5)	4 (1.2)	6 (1.8)	4 (1.2)	0.916
No photocoagulation, No. (%)	132 (41.1)	126 (39.8)	133 (40.5)	126 (39.1)	0.961
Localized photocoagulation, No. (%)	74 (23.0)	71 (22.4)	85 (25.9)	76 (23.6)	0.748
Extensive photocoagulation, No. (%)	115 (35.8)	119 (37.6)	110 (33.5)	120 (37.2)	0.690
Buckling, No. (%)	4 (1.2)	4 (1.2)	3 (0.9)	3 (0.9)	0.954
Cryopexy, No. (%)	3 (0.9)	1 (0.3)	4 (1.2)	3 (0.9)	0.650
No tamponade, No. (%)	79 (24.6)	74 (23.4)	86 (26.2)	87 (27.0)	0.724
Air tamponade, No. (%)	88 (27.4)	81 (25.6)	84 (25.6)	79 (24.5)	0.869
SF6 tamponade, No. (%)	97 (30.2)	98 (31.0)	102 (31.0)	94 (29.1)	0.949
C3F8 tamponade, No. (%)	8 (2.4)	7 (2.2)	8 (2.4)	9 (2.7)	0.973
Silicone oil, No. (%)	49 (15.2)	56 (17.7)	48 (14.6)	53 (16.4)	0.722
Length of surgery (minutes)	82 ± 27 (35–135)	81 ± 25 (40–140)	79 ± 23 (40–130)	80 ± 24 (35–140)	0.515

Footnote: SD = standard deviation; OR = odds ratio; CI = confidence interval; IOP = intraocular pressure; IOL = intraocular lens.

**Table 2:** Proportion of patients with complete response during the first 24 hours after surgery.

Treatment Group	Number of Patients	Number of Patients with Complete Response	Percentage of Patients with Complete Response
Placebo, No. (%)	321	231	71.96 (%)
Ondansetron, No. (%)	316	254 ^a^	80.38 (%)
Dexamethasone, No. (%)	328	265 ^b, d^	80.79 (%)
Ondansetron+Dexamethasone, No. (%)	322	309 ^c, e, f^	95.96 (%)

Footnote: KRUSKAL-WALLIS *p* < 0.001; Post-hoc pairwise comparison (Dunn-Bonferroni): ^a^ *p* = 0.036 placebo vs ondansetron; ^b^ *p* = 0.023 placebo vs dexamethasone; ^c^ *p* < 0.001 placebo vs ondansentron + dexamethasone; ^d^ *p* = 1 ondansetron vs dexamethasone; ^e^ *p* < 0.001 ondansetron vs ondansetron + dexamethasone; ^f^ *p* < 0.001 dexamethasone vs ondansetron + dexamethasone.

**Table 3:** Proportion of patients with postoperative nausea and vomiting (PONV) divided by gender.

Treatment Group	Number of Patients (%)	Number of Patients with PONV (%)
Placebo, No. (%)	321 160 males–161 females (49.8–50.2)	90 41 males–49 females (45.2–54.8) ^a^
Ondansetron, No. (%)	316 157 males–159 females (49.7–50.3)	62 28 males–34 females (44.9–55.1) ^a^
Dexamethasone, No. (%)	328 164 males–164 females (50.0–50.0)	63 27 males–36 females (43.3–56.7) ^a^
Ondansetron + Dexamethasone, No. (%)	322 162 males–160 females (50.3–49.7)	13 6 males–7 females (44.2–55.8)

KRUSKAL-WALLIS *p* = 0.999 among four groups; Chi square, (rate of PONV in males and in females) ^a^ < 0.001.

**Table 4:** Proportion of patients with PONV, with and without arterial hypertension.

Treatment Group	Number of Patients with PONV	Cases of PONV in Patients with Hypertension (%)	Cases of PONV in Patients without Hypertension (%)
Placebo, No. (%)	90	57 (63.5)	33 (36.5) ^a^
Ondansetron, No. (%)	62	38 (61.3)	24 (38.7) ^b^
Dexamethasone, No. (%)	63	36 (57.1)	27 (42.7) ^c^
Ondansetron + Dexamethasone, No. (%)	13	8 (61.5)	5 (38.5)

KRUSKAL-WALLIS *p* = 0.850 among four groups; Chi square, (rate of PONV in patients with and without arterial hypertension) ^a^ < 0.001, ^b^ = 0.010, ^c^ = 0.016.

**Table 5:** Standardized score of postoperative nausea and vomiting (PONV) severity and pain score.

PONV Score	Placebo (*n* = 321)	Ondansetron (*n* = 316)	Dexamethasone (*n* = 328)	Ondansetron + Dexamethasone (*n* = 322)
No PONV, No. (%)	231 (71.96%)	254 (80.38%)	265 (80.79%)	309 (95.96%)
Mild, No. (%)	62 (19.31%)	46 (14.56%)	45 (13.72%)	11 (3.42%)
Moderate, No. (%)	23 (7.17%)	16 (5.06%)	18 (5.49%)	2 (0.62%)
Severe, No. (%)	5 (1.56%)	0 (0%)	0 (0%)	0 (0%)
Pain score				
No pain, No. (%)	221 (68.8)	214 (67.7)	242 (73.7)	243 (75.4)
Mild pain, No. (%)	75 (23.3)	79 (25.0)	69 (21.0)	62 (19.2)
Moderate pain, No. (%)	23 (7.1)	21 (6.6)	16 (4.8)	17 (5.2)
Severe pain, No. (%)	2 (0.6)	2 (0.6)	1 (0.3)	0 (0%)

Standardized score of PONV severity: KRUSKAL-WALLIS *p* < 0.001; Post-hoc pairwise comparison (Dunn-Bonferroni): *p* = 0.028 placebo vs ondansetron; *p* = 0.020 placebo vs. dexamethasone; *p* < 0.001 placebo vs. ondansetron + dexamethasone; *p* = 1.000 ondansetron vs. dexamethasone; *p* < 0.001 ondansetron vs. ondansetron + dexamethasone; *p* < 0.001 dexamethasone vs. ondansetron + dexamethasone. Pain score: KRUSKAL-WALLIS *p* = 0.080.

**Table 6:** Rate of adverse events.

Non-Ocular Adverse Events	Placebo (*n* = 321)	Ondansetron (*n* = 316)	Dexamethasone (*n* = 328)	Ondansetron + Dexamethasone (*n* = 322)	KRUSKALL-WALLIS
Akathisia/Restlessness, No. (%)	0 (0%)	0 (0%)	1 (0.3%)	0 (0%)	-
Death, No. (%)	0 (0%)	0 (0%)	0 (0%)	0 (0%)	-
Dizziness, No. (%)	6 (1.9%)	4 (1.3%)	2 (0.6%)	2 (0.6%)	*p* = 0.353
Drowsiness, No. (%)	18 (5.6%)	14 (4.4%)	11 (3.3%)	21 (6.5%)	*p* = 0.270
Extrapyramidal symptoms, No. (%)	0 (0%)	0 (0%)	0 (0%)	0 (0%)	-
Headache, No. (%)	64 (19.9%)	77 (24.4%)	59 (18.0%)	73 (22.7%)	*p* = 0.209
Hypertension, No. (%)	14 (4.4%)	12 (3.8%)	16 (4.9%)	11 (3.4%)	*p* = 0.739
Myocardial infarction, No. (%)	0 (0%)	0 (0%)	0 (0%)	0 (0%)	-
Nasopharyngitis, No. (%)	9 (2.8%)	10 (3.2%)	8 (2.4%)	13 (4%)	*p* = 0.773
Stroke, No. (%)	0 (0%)	0 (0%)	0 (0%)	0 (0%)	-
Ocular adverse events					
Choroidal detachment, No. (%)	4 (1.2%)	3 (0.9%)	3 (0.9%)	2 (0.6%)	*p* = 0.878
Corneal abrasion, No. (%)	7 (2.2%)	11 (3.5%)	6 (1.8%)	10 (3.1%)	*p* = 0.524
Corneal edema, No. (%)	6 (1.9%)	9 (2.8)	13 (4%)	12 (3.7%)	*p* = 0.406
Endophthalmitis, No. (%)	0 (0%)	0 (0%)	0 (0%)	0 (0%)	-
IOP ≥ 30 mmHG, No. (%)	12 (3.7%)	8 (2.5%)	9 (2.7%)	8 (2.5%)	*p* = 0.757
Hypotony, No. (%)	4 (1.2%)	6 (1.9%)	3 (0.9%)	7 (2.2%)	*p* = 0.546
Retinal detachment, No. (%)	1 (0.3%)	0 (0%)	1 (0.3%)	0 (0%)	*p* = 0.974
Suprachoroidal hemorrhage, No. (%)	2 (0.6%)	1 (0.3%)	1 (0.3%)	0 (0%)	*p* = 0.931
Vitreous hemorrhage, No. (%)	2 (0.6%)	2 (0.6%)	5 (1.5%)	3 (0.9%)	*p* = 0.461

## Supplementary Materials

The following are available online at https://www.mdpi.com/2077-0383/8/3/391/s1: Table S1: CONSORT 2010 checklist of information to include when reporting a randomized trial.
